# Trust in the Health System and COVID-19 Treatment

**DOI:** 10.3389/fpsyg.2021.643758

**Published:** 2021-07-09

**Authors:** Armenak Antinyan, Thomas Bassetti, Luca Corazzini, Filippo Pavesi

**Affiliations:** ^1^Wenlan School of Business, Zhongnan University of Economics and Law, Wuhan, China; ^2^National Research University Higher School of Economics, Moscow, Russia; ^3^Cardiff Business School, Cardiff University, Cardiff, United Kingdom; ^4^Department of Economics and Management “Marco Fanno”, University of Padua, Padua, Italy; ^5^Department of Economics and VERA (Venice Centre in Economic and Risk Analytics for Public Policies), University of Venice “Ca’ Foscari”, Venezia, Italy; ^6^School of Economics and Management, University “Carlo Cattaneo” - LIUC, Castellanza, Italy; ^7^Stevens Institute of Technology, School of Business, Hoboken, NJ, United States

**Keywords:** COVID-19, pandemic, healthcare system, trust, survey experiment

## Abstract

COVID-19 continues to spread across the globe at an exponential speed, infecting millions and overwhelming even the most prepared healthcare systems. Concerns are looming that the healthcare systems in low- and middle-income countries (LMICs) are mostly unprepared to combat the virus because of limited resources. The problems in LMICs are exacerbated by the fact that citizens in these countries generally exhibit low trust in the healthcare system because of its low quality, which could trigger a number of uncooperative behaviors. In this paper, we focus on one such behavior and investigate the relationship between trust in the healthcare system and the probability of potential treatment-seeking behavior upon the appearance of the first symptoms of COVID-19. First, we provide motivating evidence from a unique national online survey administered in Armenia–a post-Soviet LMIC country. We then present results from a large-scale survey experiment in Armenia that provides causal evidence supporting the investigated relationship. Our main finding is that a more trustworthy healthcare system enhances the probability of potential treatment-seeking behavior when observing the initial symptoms.

## Introduction

As of April 26, 2021, there are roughly 148 million COVID-19 cases and 3.1 million deaths worldwide. Despite excessively promoted precautionary measures to seek medical attention in case of fever, cough, and difficulty of breathing, in many instances, individuals with symptoms avoid contacting health authorities. For example, a recent Gallup study finds that, in the United States, one out of every seven adults (14%) would not seek coronavirus treatment for themselves or a member of their household (Witters, 2020). There have also been repeated reports of people avoiding professional medical care in low- and middle-income countries (LMICs) lately. According to the mayor of Moscow, Sergey Sobyanin, as of April 23, 2020, roughly two-thirds of the coronavirus victims in Moscow first opted for self-care and then found themselves in the hospitals in critical condition. In India, there have been instances of isolated patients (with either confirmed or suspected COVID-19 cases) trying to run away from public hospitals (Chetterje, 2020). While in the United States the cost of medical treatment seems to be one of the main factors deterring individuals from seeking medical help (Witters, 2020), the reasons in LMICs can be considerably different. Indeed, in many of these countries, the public healthcare system bears the financial burden, treating COVID-19 for free. More specifically, in LMICs–e.g., Turkey, South Africa, India, Russia, Chile, Mexico, Colombia, Brazil–the society is plagued with a widespread trust deficit in the healthcare system because of its low quality (e.g., Ipsos, 2018; Chetterje, 2020). This trust deficit can discourage patients with COVID-19 symptoms from seeking medical care.

Patient trust in the healthcare system can be defined as the acceptance of a vulnerable situation in which the trustor (i.e., the patient) believes that the trustee (e.g., the physician) will act in the trustor’s best interest (Thom et al., 2004). In general, patient trust is firmly interconnected with the quality of the healthcare system. More specifically, physician (e.g., technical competency, interpersonal competency, vigilance, morality) and hospital attributes (e.g., valuing patient’s time, hygienic standards) serve as possible determinants of patient trust (e.g., Murray and McCrone, 2015). Thus, a healthcare system with incompetent physicians and mediocre medical institutions can largely undermine patient trust, which can shy patients away from hospitals and worsen healthcare outcomes such as chronic disease management, use of preventative services, and satisfaction with care^1^.

This paper empirically investigates the relationship between patient trust in the healthcare system and the probability of seeking professional medical help (either calling the COVID-19 hotline, or calling an ambulance, or going to the hospital) in case of first symptoms of COVID-19 in a LMIC, the Armenia. Our analysis proceeds in two steps. First, as motivating evidence, we use data from a nationwide online survey collected by the Caucasus Research Resource Centers (CRRC) to confirm the existence of a relationship between trust in the healthcare system and treatment-seeking behaviors. Then, to test the causal impact of trust on treatment-seeking behavior, we run a nationwide survey experiment in Armenia.

As an LMIC country in transition, where around half of the population is dissatisfied with the (low-quality) healthcare system (Footman et al., 2013) and one-quarter of the population does not trust doctors and nurses (Gallup, 2019), Armenia represents an ideal case for tackling the research question posed. On top of the low trust in the healthcare system, Armenia is characterized by relatively high levels of poverty (23.5%; Armstat, 2019) and corruption (Gallup, 2019). That being said, the share of Government expenditure devoted to public health in Armenia is lower than the world average (e.g., Lavado et al., 2018),^2^ which implies that the public healthcare system in Armenia is not adequately structured to fight COVID-19 once the cases increase. More specifically, the country faces a shortage of ventilators, intensive care unit (ICU) equipment, personal protective equipment, lab reagents, and supplies (Torosyan, 2020). Various international organizations support the country to address the urgent need for equipment and medical supplies^3^.

We believe that the question posed in this paper is of utmost importance for several reasons.

First, given the absence of effective antiviral treatments for COVID-19 (e.g., Richardson et al., 2020; Russell et al., 2020), the vital option to curb mortality boils down to early and strong interventions to prevent the progression of the disease (e.g., Sun et al., 2020). Second, if not treated properly at illness onset, COVID-19 can progress to a severe form. This will lead the patients to need intubation and invasive ventilation in an ICU, increasing the burden on buckling healthcare systems, considering that the world desperately scrambles for ventilators and ICU beds (Woodyatt, 2020). Third, a solid number of COVID-19 cases may remain undetected, which can contribute to the exponential transmission of the virus. Recall that rapid diagnosis, immediate isolation of cases, rigorous tracking, and precautionary self-isolation of contacts lie at the heart of effectively curtailing the outbreak of the disease (e.g., Ferretti et al., 2020; Salathé et al., 2020).

The rest of the paper is structured as follows. Section “Study Motivation” depicts the literature that motivates the study. Section “Empirical Approach” sketches the empirical approach. Section “Study 1: National Survey” describes the survey data, the empirical specification and reports the results of the estimations. Section “Study 2: The Survey Experiment” details the survey experiment, while section “Conclusion” concludes the paper and provides policy recommendations.

## Study Motivation

Our paper is motivated by three streams of literature detailed below.

The first stream explores human behavior and preferences during the COVID-19 pandemic. To this date, scholars have mainly studied social preferences during the pandemic (Branas-Garza et al., 2020), the impact of economic preferences on compliance and perception (Müller and Rau, 2020), people’s expectations about the macroeconomy (Dietrich et al., 2020; Li, 2020), citizens’ self-reported compliance and the efficacy of government communication (Barari et al., 2020), citizens’ reaction to misinformation (Bursztyn et al., 2020) and evolution of trust at different stages of the pandemic (Battiston et al., 2020). To the best of our knowledge, no paper has studied the relationship between trust in formal institutions, such as the healthcare system, and the probability of seeking professional medical treatment in case of COVID-19 symptoms.

The second stream studies the relationship between social capital, health behavior, and outcomes. Though social capital is quite a general notion (see the discussion in Hollard and Sene, 2016), we may distinguish between *horizontal (or generalized) trust*, that captures one’s trust in other members of the society, neighbors, or peers and *vertical trust*, that measures one’s confidence or trust in formal institutions (e.g., Fischer and Torgler, 2013). There is robust evidence that horizontal trust is positively related to improved health outcomes (D’Hombres et al., 2010; Ronconi et al., 2012; Herian et al., 2014; Rocco et al., 2014) and access to primary health care (e.g., Hollard and Sene, 2016) both in developed and developing countries. In the context of COVID-19, Durante and Gulino (2020) illustrate that civic culture (which includes horizontal trust) can substantially affect mobility. Similarly, vertical trust or trust in the healthcare system is shown to be linked to the use of health services, improved health outcomes, and satisfaction with care (Whetten et al., 2006; Murray and McCrone, 2015). We substantially depart from this stream of research in the sense that we investigate the connection between vertical trust and health behavior (in our case seeking for COVID-19 treatment) during a quickly evolving pandemic that created significant problems for almost everyone in the world.

The third stream discusses trust as an important factor in public compliance during a pandemic. More specifically, during the H1N1 pandemic (or the swine flu pandemic), trust in the official institutions led people to adopt recommended behaviors in Italy (Prati et al., 2011), to express intentions to get vaccinated in Netherlands (van der Weerd et al., 2011) and the United States (Quinn et al., 2009) as well as to get vaccinated in Switzerland (Gilles et al., 2011). Unlike this literature, we focus on the intentions to seek professional medical treatment in case of highly contagious and fatal disease symptoms. Furthermore, there are important differences between the H1N1 pandemic and COVID-19, which make our context different from the ones studied before. According to official WHO communication, COVID-19 is ten times deadlier than H1N1 with rather gloomy fatality forecasts (Wood, 2020). In this respect, as of April 26, 2021, around three million individuals died because of the virus worldwide. In addition, COVID-19 is estimated to be almost twice as contagious as H1N1. More specifically, while the reproduction number for H1N1 was around 1.2–1.5 (e.g., Cowling et al., 2010), the reproduction number for COVID-19 can reach up to 5.7 (e.g., Sanche et al., 2020).

## Empirical Approach

Following the findings in the medical research, we strive to test whether high trust in formal institutions, such as the government and the healthcare system, increases the probability of seeking professional medical help in case of first COVID-19 symptoms. We expect a higher probability of treatment-seeking behavior in a high-trust with respect to a low-trust environment.

To check for a relationship between trust and treatment-seeking behavior, we first utilize a nationwide online (cross-sectional) survey conducted by the CRRC from March 29 to April 8, 2020. COVID-19 is the focus of the survey and the standardized questionnaire includes seventeen questions about COVID-19, respondents’ trust in the healthcare system, and the socio-demographic profile of the respondent. The survey was the initiative of CRRC and we neither participated in the design of the questionnaire nor in the administration of the survey. We simply use the publicly available data^4^. To the best of our knowledge, this is one of the rare large-scale surveys about COVID-19 in LMICs that simultaneously elicits respondents’ trust in the healthcare system and their intentions to seek medical help in case of coronavirus symptoms.

Nonetheless, from a methodological perspective, the cross-sectional survey we utilize only allows us to establish a potential correlation between trust in formal institutions and the probability of seeking professional medical help in case of COVID-19 symptoms. In other words, the correlation between trust and the treatment-seeking behavior can be interpreted in two ways. First, the higher the trust in the healthcare system the higher the probability to seek professional medical help. Second, if a person intends to seek professional medical help, it can affect her trust in the healthcare system. To further investigate whether trust in the healthcare system affects the probability of seeking medical help, we therefore ran a nationwide survey experiment in the Armenia from April 30 to May 1, 2020^5^. While, the traditional survey does not allow us to claim any causal relationship between trust in the health system and treatment-seeking behavior, the survey experiment (and experiments in general) has two important features permitting a causal interpretation of the results:

(i) Individuals are randomly assigned to two or more groups, where one group is usually a control group. This implies that if the randomization process is successful, on average, the individuals will share similar observed and unobserved characteristics across groups.
(ii) Given a successful randomization, by providing different treatment stimuli to the groups, the researchers can attribute the observed variation in the outcome across the groups to the treatment stimuli^6^.

On top of allowing us to establish a causal relationship between trust and the probability of seeking medical care, survey experiments offer the possibility to frame the decision task by referring to the COVID-19 context and manipulate the level of trust in the healthcare system with *ad hoc* vignettes. Vignettes are short, systematically varied descriptions of situations or persons to elicit the beliefs, attitudes, or behaviors of respondents with respect to the presented scenarios (Alexander and Becker, 1978; Steiner et al., 2016). In our setting, we present vignettes that portray either a well-functioning or a malfunctioning healthcare system. Having done so, we first check whether these vignettes affect the trust of individuals in the health system. Next, we test the impact of these vignettes on the treament-seeking behavior during COVID-19. A crucial advantage of vignette studies is that a vignette can narrow the gap between the study and the real world, mimicking actual decision tasks and situations (Hainmueller et al., 2015). Indeed, this is highly relevant for our setting, in which we strive to manipulate the level of trust in the healthcare system as realistically as possible.

Even though survey experiments are a potent instrument for establishing a causal link between trust and the probability of potential treatment-seeking behavior during COVID-19, they can trigger critical concerns of external validity. For example, the results may be prone to a hypothetical bias, in the sense that the responses to the hypothetical scenarios may considerably conflict with real-world behavior. Alternatively, the specific wording of the vignettes may bias the results. In this regard, Hainmueller et al. (2015) prove the external validity of survey experiments, illustrating that the causal effects obtained in a vignette study are consistent with those of natural experiments.

## Study 1: National Survey

The online survey was circulated through Facebook ads from March 29 to April 8, 2020 and targeted adults living in the territory of Armenia. Nonetheless, it is important to note that the sample is not representative of the Armenian population. Overall, 8,427 individuals completed the questionnaire. We dropped those participants who did not answer the questions of interest (please refer to sub-sections “Statistical Analysis and Results” for more details). Altogether, we were left with 6,413 observations for the statistical analysis.

Regarding the situation of the pandemic in Armenia, the government declared the state of emergency on March 16, 2020, and the entire population was put on a strict lockdown on March 24 (though there were immense problems with enforcing the lockdown). During the survey administration the number of confirmed cases increased from 424 on March 29 to 881 on April 8. As of April 8, there were only 9 deaths registered. In case of symptoms, individuals are advised to isolate and seek professional medical care. As of May 19, all individuals with suspected and confirmed COVID-19 cases were subject to hospitalization and isolation.

### Statistical Analysis

We estimate the following regression model:

(1) $$Y_{\text{i}}=\beta_{o}+\beta_{1}\times Trust_{\text{i}}+\beta_{2}\times X_{\text{i}}+\varepsilon_{\text{i}}.$$

The dependent variable, *Y*_i_, is individual *i*’s self-reported first action in case of COVID-19 symptoms elicited through the following survey question: “*What would your first action be in case of symptoms resembling those of COVID-19 (fever, cough, sore throat, breathing difficulties, weakness)?*” To facilitate the analysis, we group the responses into two categories:

(i) Treatment-seeking behavior, if the respondent indicates that she will either call COVID-19 hotline, or call an ambulance, or go to a medical institution, or ask for help from a doctor she knows personally. In this case, the dependent variable *Y*_i_ equals 1.
(ii) Treatment-avoiding behavior, if the respondent indicates that she will either treat herself, or isolate at home and wait for recovery, or do nothing. In this case, the dependent variable *Y*_i_ equals 0.

*Trust*_i_ indicates individual *i*’s trust in the healthcare system. Specifically, the trust variable is built upon respondents’ answers to the question: ‘‘*Given the State of emergency in Armenia, how much do you trust the following institutions? Rate on a scale from 1 (Completely mistrust) to 5 (Completely trust).*’’^7^ The healthcare system is in the list of institutions the respondents had to state their trust in. *X*_i_ is a matrix containing variables about the demographic and socio-economic conditions of the respondents, such as age, income, education, and gender.

Taking the binary nature of the dependent variable into account, we estimate (1) utilizing linear probability and probit models^8^. For probit models, the marginal effects are reported. Since the responses within regions can be somewhat correlated, we cluster the standard errors at the regional level^9^.

### Results

We start the section by detailing the variables used in the analysis and providing brief descriptive statistics of the sample in Table 1.

**TABLE 1:** Variables and descriptive statistics.

Variable	Description	Frequency, Mean and Standard Deviation^∗^
**Dependent variable**		
Treatment-seeking behavior	“*What would your first action be in case of symptoms resembling those of COVID-19 (fever, cough, sore throat, breathing difficulties, weakness)?*” =1 if the respondent indicates that she will either call COVID-19 hotline, or call an ambulance, or go to a medical institution, or ask for help from a doctor she knows personally; =0 otherwise.	5676/6413 (88.508%)
**Independent variables**		
Trust in the healthcare system	Given the current state of emergency in Armenia, tell me please to what extent you trust the following institutions on a scale from 1 (Fully distrust) to 5 (Fully trust). Healthcare system	4.206 (1.098)
Age	An integer, indicating the age of the respondent.	32.360 (10.444)
University education (completed or incomplete)	=1 if the respondent received either incomplete or complete university education, i.e., completed a bachelor’s, master’s, or a Ph.D. degree; =0 otherwise.	5002/6413 (77.998%)
Vocational education	=1 if the respondent received vocational education;=0 otherwise.	777/6413 (12.116%)
School diploma or lower (the omitted category)	=1 if the respondent received either no formal education, or primary education (either complete or incomplete) or secondary education (either complete or incomplete);=0 otherwise.	634/6413 (9.886%)
Male	=1 if the respondent is male; =0 otherwise.	2075/6413 (32.356%)
Low-income group (the omitted category)	=1 if the respondent’s reported income is in one the following income categories: up to 24,000 AMD^10^; 24,001–48000 AMD; 48,001–120,000 AMD;=0 otherwise.	2876/6413 (44.846%)
Medium-income group	=1 if the respondent’s reported income is in one of the following income categories: 120,001–192,000 AMD; 192,001–383,000 AMD; 383,001–575,000 AMD;=0 otherwise.	3140/6413 (48.963%)
High-income group	=1 if the respondent’s reported income is in one of the following income categories: 575,001–969,000 AMD; more than 969,001 AMD;=0 otherwise.	397/6413 (6.191%)

The description of the variables used in the analysis. *In case of Trust in the healthcare system and Age the mean and the standard deviation (in the parentheses) are provided.

Overall, around 32.4% of the sample is male. The mean respondents’ age is 32.4 years old. Roughly 76.8% of the respondents have either a complete (62.4%) or incomplete (14.4%) university education. Given that only 20% of the population has higher education in Armenia, according to the results of the 2011 population census (Armstat, 2013), we are dealing with an educated sample. This is not much of a surprise since educated individuals with high income are usually overrepresented in online surveys. Regarding the first response to COVID-19 symptoms, the majority of the respondents self-report potential treatment-seeking behavior as a first behavioral response.

Table 2 collects the estimates of linear probability and probit models described in sub-section “Statistical Analysis.”

**TABLE 2:** Regression results.

	(1) LPM	(2) LPM	(3) Probit	(4) Probit
Trust in the healthcare system	0.019*** (0.002)	0.019*** (0.002)	0.018*** (0.002)	0.017*** (0.002)
Male		−0.012 (0.007)		−0.012* (0.007)
Vocational education		−0.015 (0.016)		−0.014 (0.016)
University education (completed or incomplete)		−0.011 (0.014)		−0.011 (0.015)
Medium-income group		0.009 (0.009)		0.009 (0.009)
High-income group		0.007 (0.015)		0.006 (0.015)
Age		−0.002*** (0.000)		−0.002*** (0.000)
Constant	0.803*** (0.011)	0.875*** (0.012)		
*F* statistics or Wald-χ^2^	61.480	84.885	67.965	712.578
*R*^2^ or pseudo *R*^2^	0.004	0.008	0.006	0.011
Number of observations	6,413	6,413	6,413	6,413

Results from OLS (clustered standard errors in parentheses) and probit (clustered standard errors in parentheses) models. For the probit model marginal effects are reported. Significance levels: *p < 0.1; **p < 0.05; ***p < 0.01.

The positive and highly significant coefficient of *Trust in the healthcare system* indicates a positive association between trust in the respective institution and treatment-seeking behavior as a first response to COVID-19 symptoms. One possible interpretation of this correlation is that high trust increases the probability of seeking professional medical care. According to this line of reasoning, the more the patient trusts the healthcare system, the more likely she is to opt for care in case of first symptoms of COVID-19. Interestingly, we also detect a significant age effect, whereby the probability of reporting treatment-seeking behavior decreases with age.

#### Result 1

There is a positive relationship between trust in the healthcare system and self-reported treatment-seeking behavior as a first response to COVID-19 symptoms.

## Study 2: The Survey Experiment

### Design

To demonstrate the causal impact of trust on the probability of treatment-seeking behavior, we administer a survey experiment consisting of *High-Trust* (HT) and *Low-Trust* (LT) treatments. In both treatments, individuals are requested to respond to an online questionnaire that consists of two sections. The second section, kept constant across treatments, includes regular questions about the demographic and socio-economic conditions of the respondents. In the first section, we manipulate trust through vignettes, depicting a third person living in a hypothetical country with either a low- or high-quality healthcare system. This person exhibits COVID-19 symptoms and must decide between two treatment-seeking actions (either call an ambulance or go to the hospital) and three treatment-avoiding actions (either self-treatment or isolation and waiting for recovery or living an ordinary life). The respondents are asked to advise the third person to decide between these five alternatives. The responses to the vignettes constitute our main variable of interest. On top of advising the third person, the respondents can also justify their advice in a short statement.

Regarding the vignettes, there are a few design issues worth detailing.

First, the trust manipulation stems from the paper by Murray and McCrone (2015), which provides an integrative review of empirical studies on factors promoting the patient-healthcare system (provider) relationship. In general, patient’s trust is closely interconnected with the quality of the healthcare system. A healthcare system with incompetent physicians and déclassé medical institutions can largely undermine patient’s trust, which in its turn can shy patients away from clinics and worsen healthcare outcomes such as chronic disease management, use of preventative services, and satisfaction with care. That is why we manipulate trust by altering the quality of the healthcare system in the vignettes.

Second, for each vignette, we introduce a trust manipulation question to guarantee that the description of low- and high-quality healthcare systems indeed influences the level of perceived trust in the healthcare system. More specifically, before being presented with the situation of the hypothetical person and advising an action in case of COVID-19 symptoms, each participant is requested to state to what extent an individual should trust a healthcare system, described in the same conditions as in the vignette. This allows us to determine whether our results are inspired by trust (as self-reported in the trust manipulation question), rather than by other unobservable individual beliefs or perceptions^11^.

Third, a hypothetical third person residing in a hypothetical country is described in the vignettes. By using a third person, we aim to liberate the study participants from their own circumstances. We assume that the respondents will apply their own preferences when advising the third person. Projecting the vignettes onto a third person is a common approach in the experimental literature (e.g., Johansson-Stenman et al., 2002; Carlsson et al., 2007; Antinyan et al., 2020).

**FIGURE 1:**
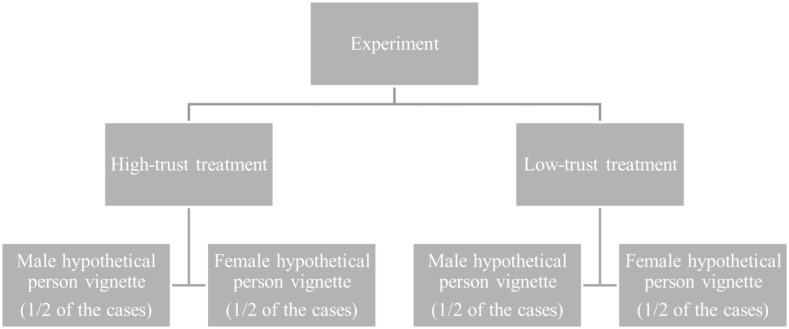
The structure of the survey experiment. The allocation to various treatments and vignettes is randomly determined by the computer.

Fourth, we change the gender in the vignettes to avoid potential interactions between the gender of the third person and the responses of the participants. In 50% of the cases, randomly selected, the hypothetical third person is a male, while in the remaining 50% of the cases, the hypothetical third person is a female. Figure 1 depicts the structure of the survey experiment, while Table A1 in Appendix A details the vignettes and trust manipulation questions.

The survey experiment was again conducted online and circulated through Facebook ads from April 30 to May 1, 2020. Like the survey, the study targeted individuals living in the territory of Armenia, though the sample is not representative of the Armenian population. As of May 1, there were 2,148 confirmed cases and 33 deaths in the country.

In total, 998 (out of 1,835) respondents completed the questionnaire. We further dropped 34 observations since the respondents indicated residence outside Armenia. An additional 16 observations were dropped because of unrealistic answers to the question about age. Altogether, we were left with 948 observations for the statistical analysis.

### Results of the Survey Experiment

We once again start illustrating the results by detailing the variables used in Study 2 in Table 3. The treatment arms are balanced with respect to observable socio-economic and demographic characteristics. Appendix B reports the balancing tests.

**TABLE 3:** Variables and descriptive statistics.

Variable	Description	Frequency, Mean and Standard Deviation^∗^
**Dependent variable**		
Treatment-seeking behavior	*Robert [Anna] has developed symptoms that resemble those of COVID-19 (coronavirus) symptoms: temperature, tiredness, sore throat, cough. In your opinion, what should Robert’s first action be?*	High-trust treatment: 440/492 (89.431%)
	=1 if the respondent indicates that she will either call emergency, or visit a medical institution;=0 otherwise.	Low-trust treatment: 295/456 (64.693%)
**Independent variables**		
High trust dummy	=1 in High-trust treatment;	492/948 (51.899%)
	=0 otherwise.	
Gender of the vignette	=1 if the gender of the third person in the vignette is male;	484/948 (51.055%)
	=0 otherwise.	
Age	An integer, indicating the age of the respondent.	35.555 (12.088)
School diploma or lower (the omitted category)	=1 if the highest level of education completed by the respondent is the school’s diploma or the respondent has no education;	263/948 (27.743%)
	=0 otherwise.	
Bachelor’s degree	=1 if the highest level of education completed by the respondent is the bachelor’s degree;	347/948 (36.603%)
	=0 otherwise.	
Master’s degree or above	=1 if the highest level of education completed by the respondent is either the master’s degree or the doctoral degree;	338/948 (35.654%)
	=0 otherwise.	
Working	=1 if the respondent is employed either full-time or part-time or self-employed;	431/948 (45.464%)
	=0 otherwise.	
Male	=1 if the respondent is male;	220/948 (23.207%)
	=0 otherwise.	
Low-income group (the omitted category)	=1 if the respondent’s reported income is in one the following income categories: up to 24,000 AMD; 24,001–48000 AMD; 48,001–120,000 AMD;	587/948 (61.920%)
	=0 otherwise.	
Medium-income group	=1 if the respondent’s reported income is in one of the following income categories: 120,001–192,000 AMD; 192,001–383,000 AMD; 383,001–575,000 AMD;	273/948 (28.797%)
	=0 otherwise.	
High-income group	=1 if the respondent’s reported income is in one of the following income categories: 575,001–969,000 AMD; more than 969,001 AMD;	88/948 (9.283%)
	=0 otherwise.	

The descriptive statistics of the variables used in the analysis. *In case Age the mean and the standard deviation (in the parentheses) are provided.

**FIGURE 2:**
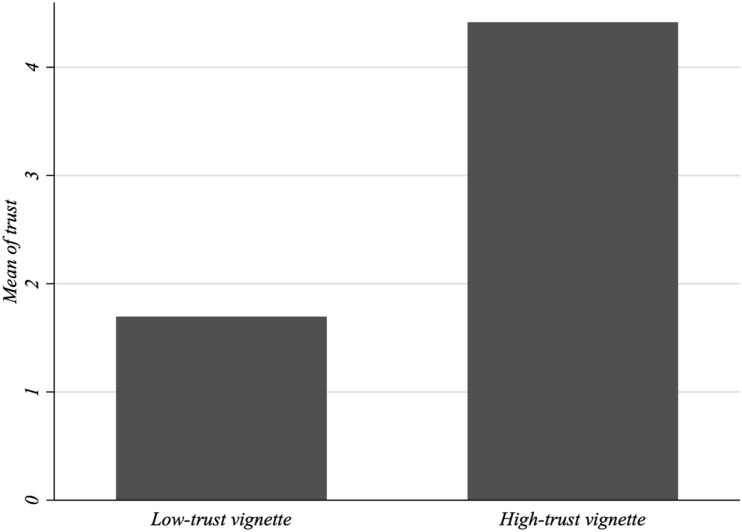
Trust manipulation in the experiment. Trust in the health system in the experiment.

Next, we report the results of the survey experiment. First, we check whether the scenarios described in the questionnaire affect individuals’ perceived trust in the health system. To do so, we compare the responses to the trust manipulation questions. According to Figure 2, trust in the healthcare system is considerably higher in the high- than in the low-trust scenario.

The non-parametric Mann–Whitney *U* test suggests that the differences in trust are statistically significant (*Z* = −24.323, *p*-value = 0.000). Thus, the scenarios effectively alter the participants’ perceived trust in the healthcare system.

After the manipulation check, we focus on the first potential actions suggested by participants in case of COVID-19 symptoms by comparing the responses to the vignettes across treatments. To facilitate the analysis, we group the responses to the vignettes into two categories:

(i) Treatment-seeking advice, if the respondent suggests either to go to a medical institution or to call an ambulance.
(ii) Treatment-avoiding advice, if the respondent suggests either to isolate at home and treat herself, or to isolate at home and wait for recovery, or to do nothing and live an ordinary life.

**FIGURE 3:**
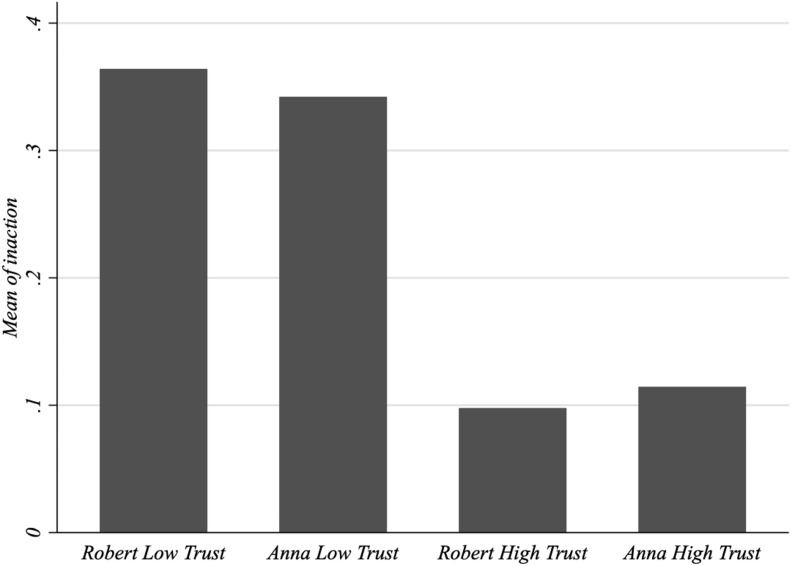
Frequency of treatment-avoiding advice. Frequency of treatment-avoiding advice in the vignettes.

Figure 3 reports the frequency of individuals who suggest treatment-avoiding behavior in the high-trust and low-trust vignettes. Remarkably, according to the figure, the frequency of suggested treatment-avoiding advice substantially differs across the high trust and low-trust vignettes.

To isolate the effect of various determinants–type of the vignette, gender of the vignette, socio-economic and demographic variables–on suggesting treatment-seeking behavior as a first response in case of COVID-19 symptoms, we utilize parametric regression techniques. We regress the respondents’ suggestions in case of COVID-19 symptoms on a *High-Trust dummy*, which equals 1 if the vignette refers to the high-trust scenario and to 0 otherwise, as well as on a set of socio-economic and demographic controls detailed in Table 3. We also account for the gender of the third person depicted in the vignettes.

Given the binary nature of our dependent variable, which equals 1 if treatment-seeking behavior is suggested and 0 if treatment-avoiding behavior is suggested, we estimate both linear probability and probit models^12^. Since the responses within regions can be somewhat correlated, we cluster the standard errors at the regional level^13^. Table 4 reports the estimates.

**TABLE 4:** Results.

	(1) LPM	(2) Probit
High-trust dummy	0.249*** (0.034)	0.248*** (0.032)
Gender of the vignette	−0.003 (0.023)	−0.002 (0.023)
Male	−0.010 (0.030)	−0.013 (0.030)
Working	0.029 (0.033)	0.026 (0.034)
Age	−0.001 (0.001)	−0.001 (0.001)
Medium-income group	−0.081** (0.030)	−0.080*** (0.027)
High-income group	−0.060 (0.042)	−0.056 (0.041)
Bachelor’s degree	−0.034 (0.038)	−0.033 (0.040)
Master’s degree	−0.020 (0.026)	−0.013 (0.030)
Constant	0.719*** (0.053)	
*F* statistics or Wald-χ^2^	774.506	56,474.465
*R*^2^ or pseudo *R*^2^	0.089	0.095
Number of observations	948	948

Results from OLS (clustered standard errors in parentheses) and probit (clustered standard errors in parentheses) models. For the probit model marginal effects are reported. Significance levels: *p < 0.1; **p < 0.05; ***p < 0.01.

The positive and highly significant coefficient of the *High-trust dummy* suggests that the probability of advising treatment-seeking behavior as a first reaction to COVID-19 symptoms is considerably higher in the high-trust vignette than in the low-trust vignette. Notice that the coefficients of the linear probability model almost coincide with the marginal effects based on probit estimates^14^. This means that the linear model is rather accurate in approximating the partial effects of the explanatory variables. Therefore, if we divide the linear coefficient of the high-trust dummy (i.e., 0.249) by the standard deviation of the dependent variable (i.e., 0.418), we have that our treatment effect is associated with a change of 59.6 standard deviation percentage points of the outcome.

We further probe the qualitative responses in the low-trust vignette to understand the justifications behind the respondents’ treatment-avoiding advice. Unfortunately, only 39 individuals who suggested treatment-avoiding advice justified their response. The majority of these justifications are indicative of lack of trust, in the sense that the patient (i.e., the trustor) does not believe that the physician or the hospital (i.e., the trustee) will act in her best interest (Thom et al., 2004). For instance, the respondents revealed the following (common) concerns:

(i) “*Since the doctors cannot be relied on, self-treatment seems to be the best option*”;
(ii) “*Human lives cannot be placed in the hands of such doctors*,”
(iii) “*If the doctors are not competent and the hospital is not sufficiently equipped it makes no sense to seek treatment*”;
(iv) “*Self-treatment, since given the description the patient would not receive sufficient treatment in the hospital*”;
(v) “*If the country lacks a good health system, that’s the only option. The health system should induce trust*”;
(vi) “*Maybe it’s also a matter of national mentality. In any case, in the given circumstances it is better to rely on the care of adequate relatives, since from a psychological perspective he/she will recover faster*.”

The complete list of responses can be found in Appendix C^15^.

Stemming from the above mentioned quantitative and qualitative considerations, we formulate the following result:

#### Result 2

High trust in the health system increases the probability of seeking professional medical assistance in case of the first symptoms of COVID-19.

## Conclusion

“*The growing COVID-19 crisis threatens to disproportionately hit developing countries, not only as a health crisis in the short term but as a devastating social and economic crisis over the months and years to come*” (UNDP, 2020). Compared to HICs, LMICs start the fight against COVID-19 from a disadvantaged position. First, under-resourced hospitals and fragile health systems will likely be quickly overwhelmed in these countries. A spike in cases because of lack of access to soap and water may complicate the situation even further in several low-income countries. Second, there is a massive distrust between citizens and formal institutions in LMICs, including the trust citizens exhibit toward the healthcare system. Low trust in formal institutions can induce the citizens to get engaged in a number of uncooperative behaviors, which can severely undermine the efforts that governments exert to stop COVID-19 in LMICs.

In this study, we explored how the degree of distrust in the public health system may play a role in dissuading citizens from actively treating themselves and, therefore, possibly adopting behavior that can facilitate the spread of contagious diseases as well as increase the chances of developing more severe symptoms. Our main finding, as implied by both the correlation analysis on nationwide cross-sectional survey data and the causal evidence drawn from the survey experiment, is that developing countries that have more trusted institutions may therefore be more likely to contain the potentially devastating effects of pandemics.

Our findings suggest that on top of implementing standard policy responses to curb the contagion such as introducing lockdowns, social distancing measures, and making face masks mandatory, governments in developing countries may need to enhance the society’s trust in the healthcare systems by improving the quality of the frontline. Given the financial constraints, policymakers may opt for interventions that increase community monitoring, which empowers patients to hold the frontline staff accountable (Björkman and Svensson, 2009; Christensen et al., 2020). Alternatively, policymakers may also try to trigger competition among clinics by introducing key performance indicators and ranking the clinics according to their outcomes (e.g., Christensen et al., 2020). Lastly, along with interventions to make the frontline more effective, policymakers may want to organize aggressive newspaper, social media, and TV campaigns to alter society’s negative image of the healthcare system and increase the uptake of the provided services. These conclusions are viable not only for the current COVID-19 epidemic but also for subsequent epidemics that will most likely occur in the future.

## Data Availability Statement

The raw data supporting the conclusions of this article will be made available by the authors, without undue reservation.

## Ethics Statement

The studies involving human participants were reviewed and approved by the Ethical Committee of the Department of Economics, Ca’ Foscari University of Venice. The patients/participants provided their written informed consent to participate in this study.

## Author Contributions

All authors listed have made a substantial, direct and intellectual contribution to the work, and approved it for publication.

## Conflict of Interest

The authors declare that the research was conducted in the absence of any commercial or financial relationships that could be construed as a potential conflict of interest.

## Footnotes

1 There is considerable evidence that social capital–proxied either by trust in the society members or by trust in formal institutions–serves as a viable determinant of health behavior and outcomes (Herian et al., 2014; Rocco et al., 2014; Murray and McCrone, 2015; Hollard and Sene, 2016).

2 In 2014, the unweighted world average was around 11.8% as compared to roughly 7% in Armenia. According to WHO estimates, such a stark difference is preserved in subsequent years as well (an interested reader can refer to WHO’s Global Health Expenditure Database (http://apps.who.int/nha/database, retrieved on May 17, 2020).

3 See e.g., https://www.worldbank.org/en/news/press-release/2020/04/03/world-bank-supports-armenia-to-combat-the-human-impact-of-the-covid19-pandemic (retrieved on May 19, 2020).

4 https://www.crrc.am/en/research/covid19-online-survey/ (retrieved on May 22, 2021). Please note that the questionnaire and the data are available in the Armenian version of the web-page. When writing this paper, we used the Armenian version of the questionnaire and translated the questions of interest into English ourselves.

5 The Ethical Committee of the Department of Economics, University of Venice “Cà Foscari” approved the experimental protocol. The participants provided their written informed consent to participate in the study.

6 For a methodological discussion about experiments an interested reader can refer to Falk and Heckman (2009), Banerjee and Duflo (2009), Glennerster and Takavarasha (2013) among others.

7 During the onset of the pandemic declaration of state emergency seemed to be one of the first steps in the fight against COVID-19 both in developed countries with advanced health systems and developing countries.

8 Logit model estimates lead to similar results and are available upon request.

9 We also re-estimate our models using White robust standard errors instead of clustering the error term. The results are qualitatively identical and available upon request.

10 At the time of the survey, 1 USD was around 490–500 AMD.

11 In the survey experiment, the trust manipulation question precedes the vignette. While we cannot exclude the fact that introducing the trust manipulation question could have exerted some (demand) effect on the subsequent COVID-19 advice, two experimental features of the design of the questionnaire were explicitly thought to minimize potential response biases. First, the manipulation check and the advice question were put in two separate and consecutive screens of the online questionnaire. This means that (i) the respondent was required to confirm the answer to the manipulation check before being presented with the advice question, and (ii) the screen with the advice question contained no reference to the answer to the manipulation check. Second, neither the description of the healthcare system in the manipulation check nor the advice vignette referred to the word “trust.” The word “trust” was only used in the question that elicits the respondents’ trust in the healthcare system described in the vignette.

12 Logit model estimates lead to similar results and are available upon request.

13 To account for the potential heteroskedasticity of the residuals, we also estimate our models using White robust standard errors instead of clustering the error term. The results remain qualitatively unchanged and available upon request.

14 We computed the probit marginal effects fixing all regressors at their mean values. Alternatively, one could consider the average marginal effects, calculating the marginal effects for each observation and then averaging these effects. However, in our case, the two approaches lead to statistically indistinguishable results.

15 We want to note that not all justifications were related to trust. Four justifications were related to concern for others in the sense that the respondents suggested the third person to stay at home not to harm others around. Six respondents suggested waiting until the symptoms worsen. This can be linked to trust, though we cannot be sure about it. Five other respondents were skeptical about COVID-19. Three remaining responses were not possible to classify. We also classified those responses in the high-trust treatment that advise treatment-avoiding behavior to understand whether the responses not related to trust are common across the two treatments. Only 13 individuals justified their treatment-avoiding advice in the high-trust treatment. Nonetheless, waiting until symptoms worsen and skepticism about COVID-19 were stated as potential reasons for avoiding treatment in this treatment as well. That said, randomization should balance the presence of such respondents across the two treatments. Thus, we believe that the treatment difference captures the pure effect of trust manipulated through the quality of the health system.

## Appendix A | Vignettes

**TABLE A1:** The trust manipulation questions and the vignettes.

Trust manipulation (asked before the low-trust vignette)
Imagine a country in which the healthcare system has the following characteristics:
• Doctors have low competence.
• Many doctors are not empathic to patients’ concerns and do not provide any encouragement. These doctors neither listen to the patient nor understand the patient’s needs.
• The medical institutions are characterized by long waiting times, and the hygiene standards are low.
• Not all patients in the hospitals are equal: there are certain privileged groups and doctors exhibit friendly attitude to them.
Using a scale from 1 to 5, where “1” means “Fully Distrust” and “5” means “Fully Trust,” please indicate to what extent should a citizen trust the healthcare system of the country described above.
**Low-trust vignette**
Imagine Robert [Anna], who lives in Country X. The healthcare system in Country X can be characterized as follows:
• Doctors have low competence.
• Many doctors are not empathic to patients’ concerns and do not provide any encouragement. These doctors neither listen to the patient nor understand the patient’s needs.
• The medical institutions are characterized by long waiting times, and the hygiene standards are low.
• Not all patients in the hospitals are equal: there are certain privileged groups and doctors exhibit friendly attitude to them.
Robert [Anna] has developed symptoms that resemble those of COVID-19 (coronavirus) symptoms: temperature, tiredness, sore throat, cough. In your opinion, what should Robert’s first action be (*)?
• Call emergency
• Visit a medical institution
• Isolate and get engaged in self-care
• Isolate and wait to recover
• Do nothing, live a normal life
*The order of the options presented to the respondents is randomized.
**Trust manipulation (asked before the high-trust vignette)**
Imagine a country in which the healthcare system has the following characteristics:
• Doctors have high competence.
• Many doctors are empathic to patients’ concerns and always provide encouragement. These doctors always listen to the patient and understand the patient’s needs.
• The medical institutions are characterized by short waiting times, and the hygiene standards are high.
• All patients in the hospitals are equal: there are no privileged groups and doctors exhibit friendly attitude to everyone.
Using a scale from 1 to 5, where “1” means “Fully Distrust” and “5” means “Fully Trust,” please indicate to what extent should a citizen trust the healthcare system of the country described above.
**High-trust vignette**
Imagine Robert [Anna], who lives in Country X. The healthcare system in Country X can be characterized as follows:
• Doctors have high competence.
• Many doctors are empathic to patients’ concerns and always provide encouragement. These doctors always listen to the patient and understand the patient’s needs.
• The medical institutions are characterized by short waiting times, and the hygiene standards are high.
• All patients in the hospitals are equal: there are no privileged groups and doctors exhibit friendly attitude to everyone.
Robert [Anna] has developed symptoms that resemble those of COVID-19 (coronavirus) symptoms: temperature, tiredness, sore throat, cough. In your opinion, what should Robert’s first action be (*)?
• Call emergency
• Visit a medical institution
• Isolate and get engaged in self-care
• Isolate and wait to recover
• Do nothing, live a normal life
*The order of the options presented to the respondents is randomized.

*The vignettes used in the survey experiment. In the vignette, where the portrayed third person is female, Anna is replaced with a male character.*

## Appendix B | Balancing Tests

**TABLE B1:** Balancing tests.

	Age	Working status	Low-income group	School education	Gender
Anna low-trust vignette	0.792 (1.163)	0.004 (0.047)	−0.123 (0.182)	0.083 (0.084)	−0.026 (0.041)
Robert high-trust vignette	1.085 (1.076)	0.002 (0.045)	−0.301* (0.176)	0.127 (0.081)	−0.053 (0.039)
Robert low-trust vignette	−0.406 (1.075)	0.040 (0.046)	−0.059 (0.179)	0.139* (0.083)	−0.077** (0.039)
Constant	35.173*** (0.770)	0.443*** (0.033)	3.355*** (0.130)	3.013*** (0.061)	0.272*** (0.030)
*F* statistics	0.812	0.341	1.140	1.153	1.449
*R*^2^	0.002	0.001	0.004	0.004	0.005
Number of Observations	948	948	948	948	948

**p < 0.1; **p < 0.05; ***p < 0.01.*

## Appendix C | Justification of the Responses

**TABLE C1:** Classification of the responses.

Response	Classification
Self-isolate and try to figure the problem with the ambulance.	Unclear
Nothing will happen to him/her.	Skeptic
Since the doctors cannot be relied on, self-treatment seems the best option.	Trust
Human lives cannot be trusted to such doctors.	Trust
One cannot speak on behalf of Robert without knowing him.	Unclear
Not to infect others.	Concern for others
Wake up, take care of your body hygiene: brush your teeth, rinse the throat, drink tea. Do not be afraid of anything, keep hygiene and live your daily life.	Skeptic
The best option in the given situation.	Unclear
If the doctors are not competent and the hospital is not sufficiently equipped it makes no sense to seek treatment.	Trust
Seek treatment in case of worsening symptoms.	Wait
Go to the doctor in case of worsening symptoms.	Wait
If Anna got an ordinary flu, the likelihood of getting sick with COVID-19 in the hospital will only increase.	Wait
It does not make sense to go to hospital in these circumstances.	Trust
This option is the most appropriate among all. There is no capacity in the hospital in case of the second option. In case of the third option people around are under risk. The last option would be more appropriate if there were many medical institutions. In the meantime, self-treatment without knowledge is useless.	Trust
Since COVID-19 is fake. I would respond differently, if all these were true.	Skeptic
Given the circumstances, Anna’s condition will get even worse in the hospital.	Trust
If a treatment for this illness does not exist it does not make sense to seek treatment, especially when the doctors are of such quality. Better to self-treat oneself at home.	Trust
Self-treatment: given the description, the patient would not receive sufficient treatment in the hospital.	Trust
Given the circumstances it does not make sense to go to the doctor. The most sensible option is at least to self-isolate not to harm others around.	Trust
If the health system is in such a horrible state, then it makes no sense to seek treatment. Furthermore, everyone understood that everything happening around is 90% fake. Something else is masked under this and people are being cheated.	Trust
I am skeptical about all this.	Skeptic
He/she should rely on himself/herself.	Trust
It can be a simple flu.	Wait
If the country lacks good health system, that is the only option. The health system should induce trust.	Trust
Maybe it is also a matter of national mentality. In any case, in the given circumstances it is better to rely on the care of adequate relatives, since from psychological perspective he/she will recover faster.	Trust
If he/she must queue and there is a chance that he/she is sick, he/she can infect others. In these circumstances, one should self-isolate not to infect others.	Concern for others
I think, first one should self-isolate himself/herself, if there are no serious symptoms.	Wait
Not to infect others.	Concern for others
Wait for worsening of the situation.	Wait
If the hospitals are in abovementioned condition, it is better to self-isolate.	Trust
Trust in self-isolation.	Trust
At least people are more caring at home.	Trust
Self-isolate, so that others do not get sick.	Concern for others
If Robert does not trust doctors, he can at least ask advice from relatives. He can also use literature and social media.	Trust
Since there is no such thing as COVID, I think he can live an ordinary life.	Skeptic
The most important thing during COVID is cleanness and the hygiene rules. If those are missing in the hospitals, self-isolation becomes more important.	Trust
Given the circumstances going to hospital is more dangerous.	Trust
If the doctors are incompetent and hygiene rules are not followed in the hospitals, going to hospitals is riskier.	Trust
It is better to die doing nothing than because of such doctors.	Trust
